# Characterization and structural basis for the brightness of mCLIFY: a novel monomeric and circularly permuted bright yellow fluorescent protein

**DOI:** 10.21203/rs.3.rs-4638282/v1

**Published:** 2024-07-19

**Authors:** Him Shweta, Kushol Gupta, Yufeng Zhou, Xiaonan Cui, Selene Li, Zhe Lu, Yale E. Goldman, Jody A. Dantzig

**Affiliations:** 1 Pennsylvania Muscle Institute, University of Pennsylvania, Philadelphia, PA-19104, United States of America.; 2 Department of Biochemistry & Biophysics, Perelman School of Medicine, University of Pennsylvania, Philadelphia, PA-19104, United States of America.; 3 Center for Engineering Mechanobiology (CEMB), Perelman School of Medicine, University of Pennsylvania, Philadelphia, PA-19104, United States of America.; 4 Department of Physiology, Perelman School of Medicine, University of Pennsylvania, Philadelphia, PA-19104, United States of America.; 5 Present address: Departments of Pharmacology and Cellular and Molecular Biology, University of California, Davis, CA-95616

**Keywords:** Fluorescent proteins, circular permutation, X-ray crystallography, small-angle X-ray scattering, analytical ultracentrifugation, fluorescence lifetime, fluorescence resonance energy transfer

## Abstract

We present mCLIFY: a monomeric, bright, yellow, and long-lived fluorescent protein (FP) created by circular permutation of YPet, the brightest yellow FP from *Aequorea Victoria* for use in cellular and *in vitro* single molecule studies. mCLIFY retains the enhanced photophysical properties of YPET as a monomer at concentrations ≤ 40 μM. In contrast, we determined that YPet has a dimerization dissociation constant (*K*_D_^1–2^) of 3.4 μM. Dimerization of YPet can cause homo-FRET, which underlies quantitative errors due to dimerization and homo-FRET. We determined the atomic structure of mCLIFY at 1.57 Å resolution and used its similarity with Venus for guided chromophore-targeted substitution studies to provide insights into its enhanced photophysical properties. The mutation V58L within the chromophore pocket improved quantum yield and extinction coefficient, making mCLIFY ~30% brighter than Venus. The extensive characterization of the photophysical and structural properties of YPet and mCLIFY presented here allowed us to reveal the basis of their long lifetimes and enhanced brightness and the basis of YPet’s dimerization.

## Introduction

Advances in biological research requiring more precise tools has led to the development of fluorescent proteins (FP) with novel photo- and biophysical properties^1–3^. FPs with varying spectral properties and the capability to report on many different cellular activities are revolutionizing the study of biological systems^4–7^. Thus, we sought to create a bright, long lived and monomeric YFP with less extensible termini for use as a donor or acceptor in a fluorescent force sensor by implementing a comprehensive approach that integrates structural, solution biophysical, and photophysical techniques.

YPet (**Y**ellow fluorescent **P**rotein for **e**nergy **t**ransfer)^8^, the brightest FP from *Aequorea victoria*^9^, was derived through a series of mutagenesis and screening experiments using the yellow fluorescent protein (YFP) variants YFP3^8^ and Venus^10^. YPet has been widely used in FRET (**F**luorescence **R**esonance **E**nergy **T**ransfer), force-FRET^11^, fusion-protein^12^, and localization studies^13^. However, its oligomeric properties are problematic. The self-association leads to homo-FRET^14^ and complicates the quantification and interpretation of biophysical signals in single molecule studies. Previous high-throughput screening approaches utilizing FPs for the directed evolution of FRET-sensors have resulted in better energy transfer signals^8^. However, this improvement may be due to the formation of intramolecular complexes, as many FPs^15^, like YPet, show weak dimerization. Additionally, oligomerization of FPs can cause experimental artifacts under overexpression conditions such as erroneous cellular localization and false positives in resonance energy transfer assays^8,15^. Several attempts have been made to eliminate YPet’s self-association, but these efforts have resulted in poor photostability^16^ or dimmer variants^17^.

A major obstacle to further improvement of YPet using rational engineering to create a monomeric derivative, has been the absence of high-resolution structural information to reveal the molecular basis for its significantly improved properties (quantum yield (QY), extinction coefficient (*ε*_max_), molecular brightness and fluorescence lifetime (τ) over its parental FPs, YFP3 and Venus. While the atomic structure of Venus (PDB ID: 1MYW)^18^, is available, it alone does not provide the structural information required to understand the improvements in YPet.

Here, we present mCLIFY (**m**onomeric, **C**ircularly permuted, long **L**ifetime, **I**ntense, **F**luorescent **Y**ellow protein): a novel and robust FP created via circular permutation of the YPet amino acid sequence, and engineered to abrogate oligomerization by mutating its predicted dimerization interface. mCLIFY retains YPet’s favorable spectral and photophysical properties, while still being monomeric at ≤ 40 μM. To learn the molecular bases for mCLIFY’s inherited photophysical properties, compared to other YFPs alone and in FRET-based biosensors, we determined its atomic structure. Furthermore, the similarities and differences between the sequences of Venus and mCLIFY guided us to perform mutagenesis to identify the amino acids, individually or together, that influence the improved photophysical properties. mCLIFY, is the brightest monomeric FP derivative, which originates from *Aequorea Victoria*, making it an advantageous choice for localization, fusion protein, and FRET-biosensor studies with single molecule resolution.

## RESULTS

### Design of mCLIFY:

**Figure 1:**
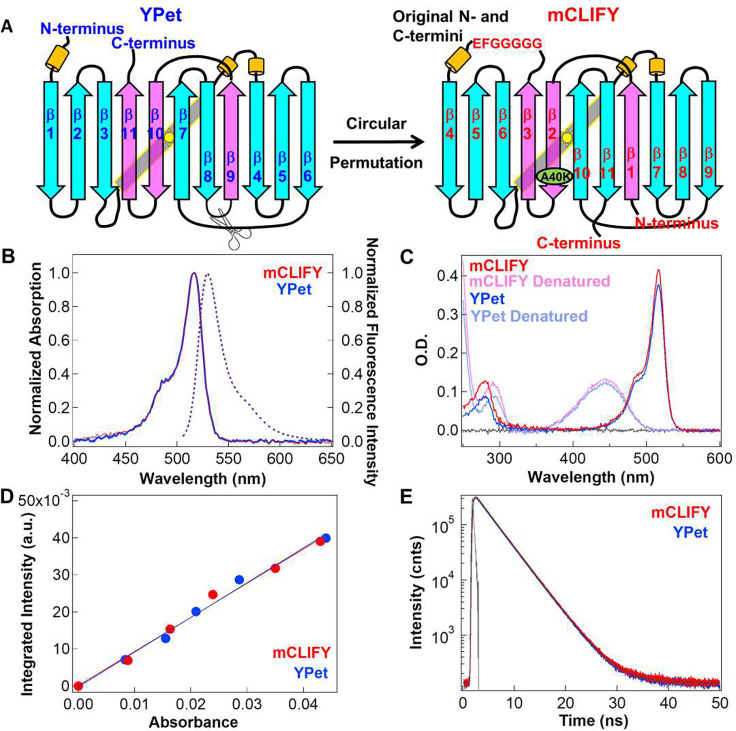
Fluorescent protein design, spectral and photophysical properties. (A) Design: Cartoon illustrating the circular permutation of YPet via severance of the loop between β- strands 8 and 9 to create new and less compliant N- and C- termini and ligating the original N- and C-termini with 7 a.a. to construct mCLIFY. The β- strands in cyan (residue 1–174) and pink (residues 175–245) specify the sequences that are reordered. The point mutation A40K to disrupt dimerization in mCLIFY is shown in green. The yellow cylinders represent alpha helices. (B) Spectral Properties: Absorption (solid line) and emission (dashed line) spectra of mCLIFY (red) and YPet (blue). Samples were excited at 488 nm to measure the emission spectra. (C) Extinction coefficient measurement: Absorbance spectra of mCLIFY and YPet under native (dark red and blue) and denatured (pale red and blue) conditions (Methods). (D) Quantum yield: The fluorescence intensities for mCLIFY (red) and YPet (blue) integrated from 500–700 nm ploted against the protein’s absorbance. Quantum yeild was calcuated from the slope of the linear regression line. (E) Time-resolved fluorescence decay of mCLIFY (red) and YPet (blue) fitted using two exponentials.

To create mCLIFY, the amino acid sequence of YPet was circularly permuted^19^. We joined the N- and C-termini of YPet via a seven amino acid linker and formed new termini by severing the short amino acid loop between β-strands 8 and 9 at the opposite end of the β-barrel (Fig. 1A, Supplementary Information (SI) Fig. S1). We expected this modification to decrease the compliance of the native N- and C-termini^11^, while preserving the native environment of the central chromophore and canonical fold.

To prevent self-association observed with YPet, we mutated its most likely dimer interface predicted based on the structure of Venus. Venus and YPet share 94% sequence identity, differing by only six amino acids. The residues that form the interface in Venus and YPet are nearly identical. Venus is also known to form dimers in solution with *K*_D_^1–2^ = 0.19 mM,^20^ while mCLIFY remains monomeric at concentrations ≤ 40 μM (see below). Prior mutagenesis studies of Venus and structural comparison with known dimeric FPs^18^ led us to mutate a single amino acid in the hydrophobic interface between the antiparallel β-strands 9 and 10 at Venus positions Ala206, Leu221 and Phe223. The mutation of A206K in Venus or YPet, corresponding to A40K in mCLIFY, has been known to prevent dimerization.^21^ The resulting mCLIFY construct folded and matured well within 15 minutes following the initiation of transcription in bacteria at 25°C, behaviors similar to other circularly permuted proteins.^22^

### Characterization of photophysical properties:

Circular permutation can affect the spectral properties of the FPs. To characterize the photophysical properties of mCLIFY, we compared them to those of YPet. While the absorption and emission spectra of mCLIFY and YPet (Fig. 1B) were indistinguishable, with maximum absorption at λ_abs_ = 517 nm and emission at λ_em_ = 530 nm in phosphate-buffered saline (PBS) at pH 7.4, our circular permutated design had a slight enhancing effect on properties associated with the brightness of mCLIFY.

The brightness of mCLIFY as calculated by the product of the *ε*_max_ determined at λ = 517 nm and QY was slightly enhanced compared to that of YPet. *ε*_mCLIFY_ = 137,600 M^−1^ cm^−1^ ± 2,500 (mean ± SD; n = 6, Fig. 1C) was slightly higher than *ε*_YPet_ = 132,500 M^−1^ cm^−1^ ± 2,200 (mean ± SD; n = 6). The quantum yield of mCLIFY (QY_mCLIFY_) 0.76 ± 0.01 (mean ± SD; n = 4) matches the published value for YPet^8^ (QY_YPet_ = 0.77, Fig. 1D), which is on the high end of the quantum yields reported for green-yellow FPs (e.g. YPet; SI, Table 1).

There is a direct relation between QY and τ of a FP’s chromophore. The lifetime for YPet *in vitro* was unknown. We determined τ for YPet and mCLIFY in solution at nanomolar concentrations by fitting the time course of the **T**ime **C**orrelated **S**ingle **P**hoton **C**ounting decays (TCSPC; Methods). The fluorescence lifetimes of YPet and mCLIFY were indistinguishable: 3.44 ns ± 0.03 versus 3.44 ns ± 0.02 (mean ± SD; n = 6 in each case, Fig. 1E). Furthermore, lifetimes barely changed when crowding agents, such as PEG-3350 or PEG-8000, were introduced into the solution to mimic the cellular environment, or ionic strength was varied. The lifetimes showed only a ~5% decrease with increasing concentrations of PEG species (Fig. S2A, S2B) or salt (KCl or NaCl; Fig. S2C, S2D). To check if these small observed changes stemmed from changes in refractive index (*n*_*r*_), we measured the concentration dependence of *n*_*r*_ for each solution using a Goldberg-type clinical optical refractometer. As expected from the Strickler-Berg equation^23^, the relation between τ^−1^ and *n*_*r*_*²* is practically linear for both types of solution (Fig. S2). The slopes of these plots are consistent with the observed changes of τ under both conditions are primarily due to changes in *n*_*r*_.

FPs in the cellular environment can encounter a range of experimental pH conditions from 4.5 to 8. Both mCLIFY and YPet showed a decrease in absorbance and fluorescence intensity as the pH ranged from 3 to 10. The steepest part of the curve occurred between pH 4 and 7.5, yielding a half-maximal fluorescence at pH 5.52 ± 0.06 with Hill coefficient (*n*_*H*_) = 1.14 ± 0.17 for mCLIFY and pH 5.47 ± 0.04, *n*_*H*_ = 1.00 ± 0.08 for YPet (mean ± SD, n= 4; Fig. S3). To determine if the decrease of fluorescence intensity observed at low pH values was due to the low pH condition or protein denaturation, we measured and compared the fluorescence intensity of the FPs at pH ~7.4, 5.0 and at a pH titrated from 5.0 back to 7.4. The intensity decrease observed at pH 5.0 recovered by 80–90% in less than a minute when the pH was titrated back to ~7.4. The residual loss of intensity was not further recovered by waiting longer and was presumably lost due to protein quenching or denaturation.

The resistance to photobleaching for the two FPs was also very similar (Fig. S4). The average measured bleaching half-life obtained for mCLIFY was 11.62 ± 1.74 s (mean ± SD, n= 5) and for YPet was 11.81 ± 1.14 s (mean ± SD, n = 5) at 20 mW/mm^2^ in solution. In summary, the photophysical properties of YPet were maintained in mCLIFY, and in some respects, modestly enhanced by circular permutation.

### Crystal structure of mCLIFY:

**Figure 2:**
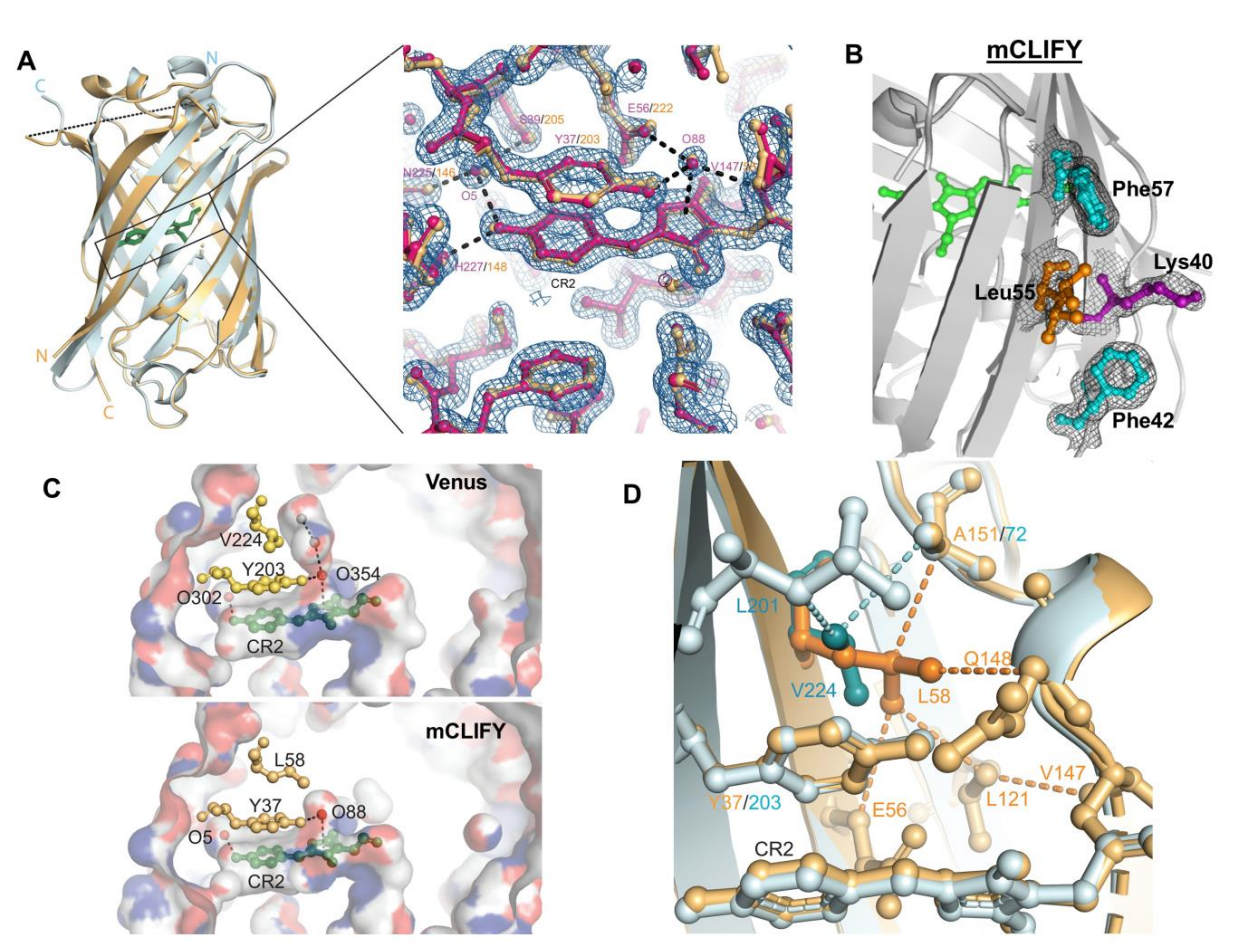
Crystal structure of mCLIFY. (A) The structure of mClify (light orange) and Venus (light cyan) are aligned and shown in cartoon representations. The N and C termini are labeled in corresponding colors. The chromophores are shown as stick representations (green). The disordered linker region of mCLIFY is shown as a dashed line. Inset: The chromophore (CR2) region of mCLIFY (magenta) is shown in ball-and-stick representation with *2F*_*o*_*-F*_*c*_ electron density map contoured at 2 *σ* (blue mesh) to validate the structure. The Venus structure, aligned with mCLIFY for comparison, is colored orange. Two water molecules (O5 and O88, spheres) are within hydrogen bond distance (dashed lines) to the chromophore and surrounding amino acids (labeled in corresponding colors). (B) Amino acids at the surface region equivalent to the dimer interface of Venus were well resolved. *2F*_*o*_*-F*_*c*_ electron density map (black mesh) contoured at 2 *σ* was shown to validate the structure. Leu55 (orange) was built as two alternate conformations. (C) Structure of the chromophore pocket. Venus (top) and mCLIFY (bottom) are shown in surface representations with atom N, O and C colored in blue, red and grey, respectively. The chromophore (CR2), the tyrosine forming π-π interaction with the chromophore, and Val224/Leu58 interacting with tyrosine are shown as ball-and-stick. Two structured water molecules are shown as red spheres. Two water molecules modeled based on the super high-resolution structure FOLD6 (PDB:7UGT), are shown as grey spheres. Hydrogen bonds are shown as dashed lines. (D) Mutation of Valine to Leucine creates new van der Waals contacts. The structures of Venus (light cyan) and mCLIFY (light orange) are shown as cartoon representations. The van der Waals contacts between L58 in mCLIFY (orange) or V224 in Venus (dark cyan) and their surrounding amino acids are shown as dashed lines between ball-and-stick representations.

To understand the molecular bases for the improved photophysical properties of YPet retained by mCLIFY requires structural information which has not yet been characterized. While our attempts to crystallize YPet failed to yield diffraction-quality crystals, we were able to generate crystals of mCLIFY that diffract to 1.57 Å resolution and solved the structure by molecular replacement (Fig. 2A, S5A, SI Table 2). Each asymmetric unit of the crystal contained one monomeric mCLIFY molecule. Electron density maps showed well-defined, continuous electron density for the canonical 11-stranded β-barrel that surrounds the chromophore. The linker introduced to connect the original N- and C-termini was absent and presumably disordered. On a monomer basis, the overall structure of mCLIFY is nearly identical to that of Venus (PDB: 1MYW)^18^. The root-mean-square deviation (RMSD) between Cα atoms of these two structures is 0.2 Å, within the error associated with this resolution. This confirms that circular permutation did not alter the canonical architecture of this FP variant.

The surface of the barrel-like structure that was targeted to impede the association between two mononers of mCLIFY was well resolved in the structure, including residues Lys40 and Phe42 (Fig. 2B). The well-defined side chains of these two residues sterically hinder appropriate dimerization by preventing a direct Van der Waals contact between a pair of Leu55 side chains from two monomers (Fig. S5B, S5C). A direct contact between those of the corresponding Leu221 residues appears to be a critical hydrophobic interaction that stabilizes the dimer interface of Venus.

The chromophore and the two highly ordered structural water molecules (O5 and O88) that directly coordinate with it were not significantly different between mCLIFY and Venus (Fig. 2A, inset). The stacked π-π interaction between the phenolic moiety of the chromophore and the side chain of Tyr37 were preserved with slight changes in orientation. The π-π stack is more parallel in mCLIFY (5° rotation between the ring systems) compared to Venus (11.3° rotation angle, Fig. 2A inset). The distal hydroxyl on the phenolic moiety that coordinates O5, the side chains of Ser39, His227, and the backbone carbonyls of Asn225 (Fig. 2A), along with the polar-π interaction between O88 and the imidazole ring of the chromophore were preserved. This latter interaction is stabilized by hydrogen bonds to the side chains of Glu56 and Tyr37 and the backbone atoms of Val147. Overall, the difference between mCLIFY and Venus are within experimental error, except near the chromophore.

Leu58 and Val147 in mCLIFY differ from Venus and are likely to influence the chromophore (Fig. S5D). The backbone of Val147 is observed to be directly connected to the chromophore, with its side chain pointing away and Leu121 in close contact. Compared to Venus, with a larger leucine side chain at position 147, the smaller valine does not impart significant changes to the surrounding environment (Fig. S5D). In contrast, the large hydrophobic side chain of Leu58 in mCLIFY contacts the π-π stacking Tyr37 more extensively than Val224 did in Venus (Fig. 2C) and buries an extra ~2 Å^2^ surface area surrounding it. Additionally, the Leu58 side chain eliminated a water-filled cavity above the chromophore of Venus (Fig, 2C). Leu58 is within Van der Waals contact of the side chains of Glu56 and Leu121, and the backbone atoms of Gln148 and Ala151, creating a distinctive network of interactions with residues near the core of the barrel (Fig. 2D). Alongside Van der Waals contacts by Glu56 and Gln148 with the chromophore, Leu58 significantly limits the positioning of the chromophore. Moreover, contact with Ala151 causes Leu58 to adopt an uncommon rotamer configuration.

### Structure-guided mutagenesis to identify key residues underlying improved photophysical properties:

The YPet sequence differs from Venus by six mutations: I47L, L68V, S208F, V224L, L231E, and D234N (Fig. S6A, S6B). mCLIFY retained the sequence identity of these critical residues, in addition to the single dimer-disrupting Lys40 mutation and an Asn68 in a region of disorder (Fig. S6A, S6B). L68V and V224L are proximal to the chromophore and S208F lies at the presumed dimer interface. The remaining three amino acids are located away from both the chromophore and the dimer interface and therefore are likely inconsequential mutations. To better understand the improved photophysical properties of YPet and mCLIFY over Venus, we focused our attention on the potentially important residues that reside near the chromophore, V58L and L147V, and the residues at the dimer interface, A40K and S42F (Fig. S7A, S7B).

We determined that the dimer-disrupting A40K mutation in mCLIFY has no significant effect on its spectral and photophysical properties compared to YPet (Fig. 1B-E). To investigate the remaining residues that possibly underlie the improved photophysical properties, we first created a variant mCLIFY^Venus^ to mimic Venus, in which Phe42, Leu58, and Val147 were reverted back to the corresponding residues Ser42, Val58, and Leu147 in Venus. Then, on this mCLIFY^Venus^ background, we reintroduced each of the three investigated mutations individually. The three resulting variants are denoted as mCLIFY^Venus-F^ (S42F), mCLIFY^Venus-L^ (V58L), mCLIFY^Venus-V^ (L147V), and mCLIFY^Venus-FL^ (S42F, V58L) the double mutant to ensure that the dimer interface doesn’t interact with the chromophore (SI Table 3). The absorption spectra of all mutants were essentially identical, with a peak at 517 nm (Fig. S7C). However, the emission spectra of mCLIFY^Venus^, mCLIFY^Venus-F^, and mCLIFY^Venus-V^ showed a small broadening, but no significant difference in the emission peak at ~528 nm (S7D).

As expected, mCLIFY^Venus^ had photophysical properties like those published for Venus. On the background of mCLIFY^Venus^, the V58L substitution, alone, resulted in photophysical properties similar to those of mCLIFY with significant improvement in QY (0.73 ± 0.01, mean ± SD, n = 3) and *ε*_max_ (140,400 ± 3200, mean ± SD, n = 3) compared to mCLIFY^Venus^ itself (Extended Fig. 1A, 1B, S8; SI Table 4). This outcome is consistent with the structural features of this substitution that we have described above. The L147V substitution increased QY (0.72 ± 0.01, mean ± SD, n = 3) but it reduced *ε*_max_ (114,800 ± 2100, mean ± SD, n = 3); the net result lead to a small enhancement in the brightness. mCLIFY^Venus-FL^ showed only a slight improvement in QY (0.67 ± 0.01, mean ± SD, n = 3) but a significant increase in *ε*_max_ (138,000 ± 4300, mean ± SD, n = 3, Extended Fig. 1A, 1B, S8). The S42F substitution at the dimerization interface had no effect on QY or *ε*_max_ and thus brightness.

Fluorescence lifetime, a crucial characteristic of FPs, showed a linear positive correlation with the quantum yield of the FP’s chromophore for the mCLIFY variants (Extended Fig. 1C and S9). mCLIFY^Venus^, with a low QY, showed a fluorescence lifetime of 3.04 ± 0.06 ns (mean ± SD, n= 4), whereas the increased QY for mCLIFY^Venus-L^ and mCLIFY^Venus-V^ correlated with increased fluorescence lifetimes of 3.2 ns ± 0.04 and 3.3 ns ± 0.02, (mean ± SD, n= 3) respectively.

### Analytical ultracentrifugation analysis of YPet and mCLIFY:

**Figure 3:**
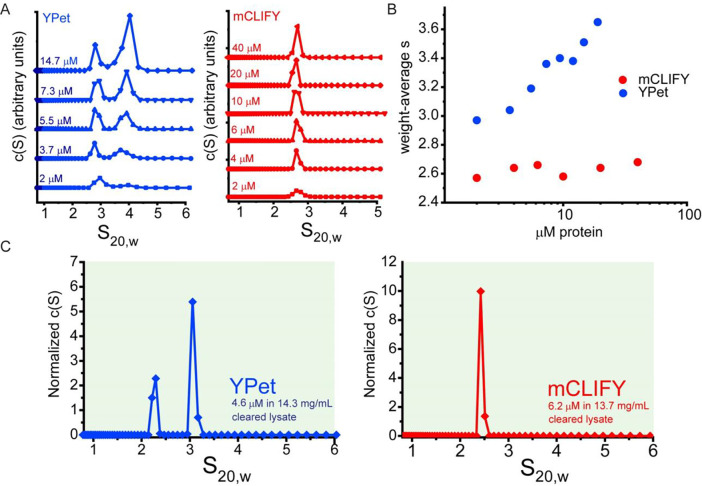
Sedimentation velocity-analytical ultracentrifugation of mCLIFY and YPet in vitro and in bacterial lysate. (A) Sedimentation coefficient distributions c(S) derived from SV-AUC data of a range of concentrations for both YPet (blue line with points) and mCLIFY (red line with points). (B) Plot of signal-weighted average S (s_w_) as a function of loading concentration, in μM monomer. Values for mCLIFY (red) and YPet (blue) at each concentration were derived from integration of the c(S) distributions shown in (A), illustrating the concentration-dependent behavior of YPet, which contrasts to the relatively consistent s_w_ determined for mCLIFY across a range of concentrations. (C) SV-AUC data at 515 nm detection were collected on bacterial lysates containing expressed YPet (4.6 μM YPet in 14.3 mg/mL cleared bacterial lysate (blue)) or mCLIFY (6.2 μM mCLIFY in 13.7 mg/ml cleared bacterial lysate (red)).

Unlike the crystallographic dimer observed with Venus, there is only one monomer of mCLIFY in the asymmetric unit, with a markedly altered dimer interface caused by the A40K and S42F mutations. It is unclear whether this interface can still mediate dimerization at the expected concentration range, besides serving as crystal lattice contacts. To assess the oligomeric state of YPet and mCLIFY experimentally, we examined them and the mCLIFY mutants using sedimentation velocity-analytical ultracentrifugation (SV-AUC)^24^. In the micromolar concentration regime, YPet displayed clear evidence of concentration-dependent self-association, with discrete concentration-dependent monomer and dimer peaks observed respectively at 2.2 and 4.7 Svedberg units (S_20,w_, mean ± SD, n = 6) in the sedimentation velocity distributions derived from the fitting the Lamm equation^25^ to data collected at various concentrations (Fig. 3A, 3B, S11 and SI Table 6). In contrast, mCLIFY appeared as a single well-defined monodispersed peak, at 2.6 ± 0.03 S_20,w_ (Fig. 3A, 3B, S10 and SI Table 5). Using a monomer-dimer association model implemented in the analysis software SEDPHAT^26^, the dissociation constant (*K*_D_^1−2^) for YPet dimerization is 3.4 ± 0.38 μM (mean ± SD, n = 5: SI Table 6). Buoyant masses estimated from the Lamm equation fits yielded 28 kDa for YPet’s monomer and ~45 kDa for its dimer, compared with 27.5 kDa and 55.1 kDa predicted from its amino acid sequence. For mCLIFY, the fitted values ranged from 27 to 30 kDa, compared with predicted 28.8 kDa. Furthermore, the mCLIFY mutants, mCLIFY^Venus^, mCLIFY^Venus-F^, mCLIFY^Venus-L^, and mCLIFY^Venus-V^, all show a well-defined single peak at 2.6 S_20,w_ at all the measured micromolar concentrations (Fig. S12), as they all contain the A40K mutation known to disrupt dimerization.

To understand the oligomeric behaviors of mCLIFY and YPet in the crowded milieu of a cellular environment, measurements were also performed in bacterial lysates from cultures expressing mCLIFY or YPet and with detection in the visible light regime. Even at the high concentration expected in the lysate, on the order of 6.2 μM, mCLIFY sedimented in a single well-defined peak in the S_20,w_ distribution, behaving like a monomer, whereas data of YPet revealed well-separated peaks corresponding to monomer and dimer species (Fig. 3C and S12). All mCLIFY mutants show a well-defined single peak at 2.6 S_20,w_ at all the measured micromolar concentrations (Fig. S13) as would be expected with the presence of the A40K mutation.

### SEC-SAXS-MALS analysis of fluorescent protein oligomeric state and conformation:

**Figure 4:**
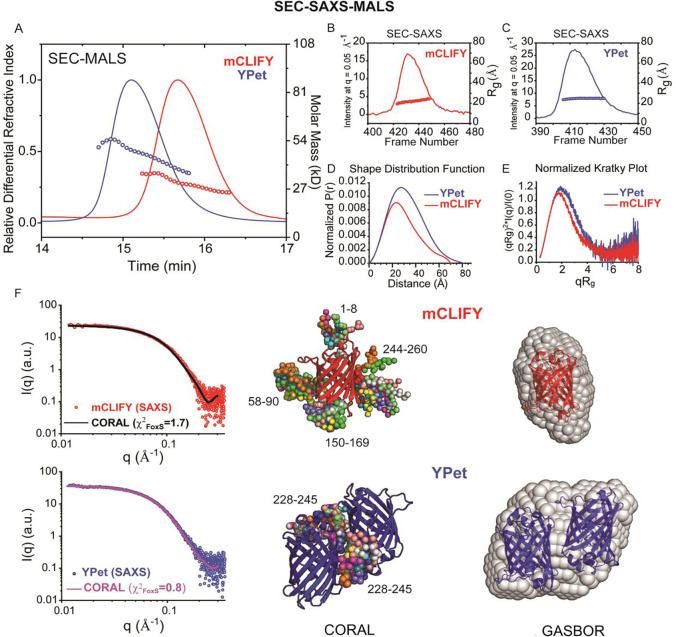
Inline size-exclusion chromatography with multi-angle light scattering detection (SEC-MALS) analyses of YPet and mCLIFY samples. (A) Shown in lines are the SEC elution profiles for YPet (blue) and mCLIFY (red), with absolute weight-averaged molecular mass (M_w_) (circles) determined by light scattering plotted across the profiles. (B) and (C) Lines show integrated intensities of small-angle scattering vs frames recorded of elution from the SEC column for mCLIFY (red, B) and YPet (blue, C). The x-ray intensity traces correspond to those obtained by UV absorption and refractive index. Circles show the derived radii of gyration (R_g_) for the background subtracted x-ray scattered profiles. (D) Shape distribution function analysis for YPet (blue line) and mCLIFY (red line), performed using the program GNOM. Parameters derived from this analysis are provided in SI Table 4. (E) Normalized Kratky Plot analysis for both YPet (blue line) and mCLIFY (red line) show characteristic bell-shaped peaks at low-*q* that returns to near-baseline at wider scattering angles, indicative of a compact, globular macromolecule. (F) GASBOR analysis: three orthogonal views of SAXS-derived shape reconstructions for monomeric mCLIFY (red, right) and dimeric YPet (blue, right) were determined by GASBOR analysis (normalized spatial discrepancy = 1.8 ± 0.14). CORAL analysis: mCLIFY (upper panels, red) and YPet (lower panels, blue) are the SAXS data derived from SVD-EFA analysis plotted on log-log scales, where intensity (*I*) is plotted vs. *q.* The solid lines are representative fits from the derived atomistic model. For mCLIFY, a superposition of ten independent calculations is shown, where the structure determined by x-ray crystallography was fixed and the amino acid regions denoted numerically were flexibly fit as beads (χ^2^_FoxS_ = 1.7). For the YPet dimer, a superposition of ten independent calculations is shown (χ^2^_FoxS_ = 0.8), where the dimer model derived from the 1MYW crystal structure was fixed and the C-terminal amino acid regions numerically denoted were flexibly fit as beads.

To further probe the oligomeric and structural properties of YPet and mCLIFY in solution, we employed size-exclusion chromatography in-line with both synchrotron small-angle X-ray scattering and multi-angle light scattering (SEC-SAXS-MALS)^27,28^. Samples of YPet and mCLIFY were injected at concentrations >200 μM, well above the *K*_D_^1–2^ for YPet dimerization determined by SV-AUC. YPet eluted earlier from the SEC column (53 μM at peak), showing a steeper sloping mass profile (from ~56 – 36 kDa) that is consistent with a monomer to dimer transition (Fig. 4A). Agreeing with the SV-AUC observations, in-line MALS analysis of the mCLIFY peak (48 μM concentration at peak by refractive index) reveals a predominantly monomeric mass profile across the peak fraction, with only a very modest change in mass profile through the ~36 – 25 kDa peak (Fig. 4A).

The sequential synchrotron SAXS scattering profiles were analyzed using singular value decomposition with evolving factor analysis (SVD-EFA)^29^. This analysis decomposes the data into their minimal components with maximal redundancy. For both YPet and mCLIFY samples, a single peak was observed when the forward X-ray scattering extrapolated to zero-angle (I(0)) was plotted versus frame number, with a consistent radius of gyration (R_g_) near ~25 Å observed across the peaks (Fig. 4B and 4C). The respective scattering profiles were assigned using mass calculations for the decomposed scattering profiles (Q_r_^30^ and Porod^31,32^; SI Table 7). For mCLIFY, only an apparent monomer species was obtained, whereas for YPet, only a dimer could be assigned. The deconvoluted SAXS profiles for both samples displayed linearity in classical Guinier analysis with R_g_s of 22.9 ± 0.2 Å and 24.8 ± 0.1 Å for mCLIFY and YPet, respectively (Fig. S14, S1 Table 7). Together with the R_g_ and maximum dimension (D_max_) determined from shape distribution (P_r_) analysis (Fig. 4D and S1 Table 7), these model-independent parameters clearly indicate significant size, shape, and mass differences between the mCLIFY and YPet preparations at the higher micromolar concentrations.

While low in resolution, SAXS analysis often allows for rigorous testing of contrasting structural models and determination of oligomeric states. The Kratky plot^31,32^ (Fig. 4E) is used to determine the degree of unfolding and/or flexibility in the samples, and Porod^31,32^ exponent analysis describes the asymptotic behavior of the intensity (*I*) as a function of scattering angle, (*q, q*_*max*_ = 0.3 Å^−1^) (SI Table 7). Both YPet (blue line) and mCLIFY (red line) show characteristic bell-shaped peaks at low-*q* that return to near-baseline at wider scattering angles, indicating that both molecules are compact in solution. Given this feature, *ab initio* shape reconstruction methods can be appropriately applied. GASBOR^33^ software predicts the overall protein structure using a chain-like ensemble of dummy residues. When tested against the SAXS data, the mCLIFY crystal structure was readily docked into its corresponding SAXS reconstruction as a monomer, with good spatial correlation (Fig. 4F, right panel) and additional space within the predicted volume rationalized as belonging to missing atomic inventory such as unresolved termini and hexahistidine tags. The GASBOR reconstruction of the YPet dimer sample, with the constraint of P2 symmetry, was readily docked with an antiparallel, side-by-side dimer configuration as observed in the crystallographic lattice of the fluorescent protein Venus (PDB ID: 1MYW)^18^. This dimeric configuration maintains the packing interaction of Ala206, Leu221, and Phe223 previously identified to underlie this dimerization interface, according to the Venus structure. Radically alternative models of dimerization, such as an elongated end-to-end arrangement, could not be accommodated by the GASBOR reconstruction.

To further correlate atomic models directly with their solution X-ray scattering, we employed the CORAL method which performs a hybrid rigid body modelling of atomic inventory with missing fragments^34^ modelled as beads. With the mCLIFY crystal structure, such modelling matched the experimental data best when N- and C- termini (a.a. 1–8 and 244–260) and loops (a.a. 58–90 and 150–169) were flexibly modelled (χ^2^_FoxS_ = 1.7–1.9 for each of ten independent CORAL calculations, Fig. 4F, left panel). In similar modelling with a Venus-derived dimer model for YPet, the unresolved C-terminal 17 residues, modelled as beads, would appear to be a more compact arrangement where the C-terminal residues pack at the dimer interface. (χ^2^_FoxS_ = 0.8–1.2 for each of ten independent CORAL calculations, Fig. 4F, left panel). Our comprehensive structural and biophysical analyses of mCLIFY and YPet show that YPet is a dimer whereas mCLIFY is a monomer even at high micromolar concentrations, validating our design rationale.

### Fluorescence anisotropy *in vitro* and in *E. coli*:

**Figure 5:**
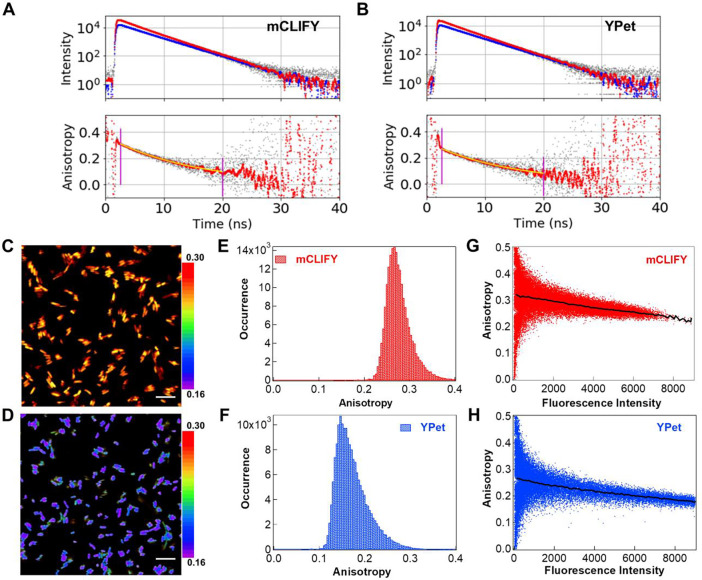
Fluorescence anisotropy of YPet and mCLIFY in vitro and in *E. Coli.* Fluorescence intensity (A, B, upper panels), parallel (red) and perpendicular (blue) to the 488 nm excitation. Time-resolved anisotropy decay curves (A, B, lower panels) for 20 nM mCLIFY and YPet in PBS buffer. The G-factor was determined by merging the tails of the decay curves. The overall average anisotropy values (<r>) are given in the text. Intensity weighted anisotropy of arctic cells expressing mCLIFY (C) and YPet (D) 45 minutes after IPTG induction (scale bar 10 μm). Intensity weighted anisotropy histograms for mCLIFY (E) and YPet (F) obtained for the entire fields shown in (C) and (D) show broad distributions with anisotropy lower for YPet than mCLIFY. (G) and (H) show the anisotropy *vs.* intensity for each cellular pixel in the images shown in figure (C) and (D).

Fluorescence anisotropy was used to investigate the oligomeric state of these fluorescent proteins (FPs)^35–37^ *in vitro* and within bacterial cells. The average anisotropy (<r>) of purified mCLIFY and YPet at nanomolar concentrations where both FPs are presumed to be primarily monomeric was 0.271 ± 0.006 and 0.239 ± 0.002, (means ± SDs, n = 3) respectively. The rotational correlation times of mCLIFY and YPet were also similar, at 15.34 ns ± 0.79 and 14.61 ns ± 0.09, respectively (means ± SDs, n = 3, Fig. 5A and 5B). This again indicates that YPet is dimeric at low micromolar concentrations.

To assess the oligomeric state at the low micromolar concentrations of many cellular studies, we investigated the concentration dependence of the time-resolved anisotropy. If mCLIFY is a monomeric protein, this anisotropy should be independent of its concentration. As predicted, the anisotropy of purified mCLIFY did not significantly change with concentration: 0.31 ± 0.006 at 0.5 μM to 0.30 ± 0.001 at 7 μM (Fig. S15A). In contrast, the anisotropy of YPet was strongly dependent on concentration, decreasing from 0.30 ± 0.008 at 0.5 μM to 0.24 ± 0.001 at 7 μM (means ± SDs, n = 3 for each, Fig. S15A). This again indicates that YPet is dimeric at these micromolar concentrations.

To mimic the cellular crowding environment, we also measured the average anisotropy of FPs at 7 μM in PBS buffer with 2.5, 5, 7.5, and 10% (w/v) polyethylene glycol (PEG-8000) (Fig. S15B). The anisotropy did not change with varying PEG-8000 concentrations for either FP. These results suggest that mCLIFY remains monomeric in the crowded environment, while YPet becomes dimeric at high concentrations.

The steady-state anisotropy of these FPs expressed in *E*. *coli* Arctic cells prior to and over a period of two hours following induction of protein synthesis was determined. The intensity-weighted anisotropy recorded for the cells 45 minutes after IPTG induction is shown in Fig 5C and 5F. The histogram illustrates increased variability and the peak shifted towards the lower anisotropy within the cells expressing YPet (Fig. 5G) compared to the cells expressing mCLIFY (Fig. 5D). Anisotropy plotted as a function of intensity in Figure 5E and 5H for each cellular pixel in the images of Figure 5C and 5F illustrates their variability within single cells and among cells in the microscopic field. The anisotropy was determined at different time intervals post induction to test the effect of increasing FP concentration over time on the anisotropy. A concentration standard curve generated with known protein concentrations in PBS buffer was used to determine the concentrations of FP in the cells (Fig S15C, see Methods). The anisotropy of mCLIFY decreased from 0.30 ± 0.005 (mean ± SD, n= 3) 15 minutes post-induction to 0.29 ± 0.02 (mean ± SD, n = 3) within 45 minutes, while the anisotropy of YPet decreased from 0.29 ± 0.003 to 0.18 ± 0.004 (means ± SDs, n = 3) in the same time frame.

### mCLIFY as a donor or an acceptor in FRET constructs:

**Figure 6:**
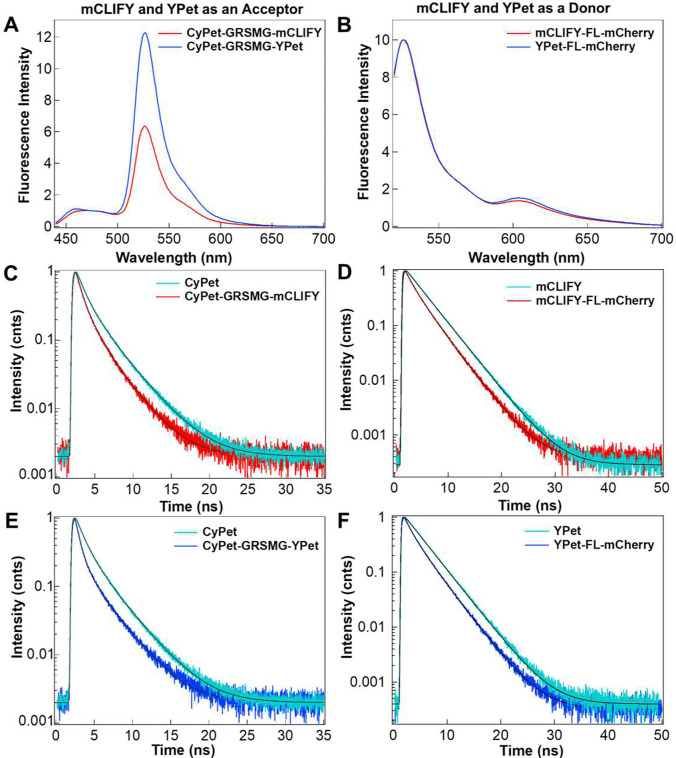
FP-FRET pair characterization (A) Emission spectra of FRET constructs in which mCLIFY and YPet are used as an acceptor. CyPet-GRSMG-mCLIFY (red) and CyPet-GRSMG-YPet (blue) were excited at 433 nm. The spectra were normalized at 475 nm to emphasize the FRET signal change. (B) Emission spectra of FRET constructs in which mCLIFY and YPet were used as donors. mCLIFY-FL-mCherry (red) and YPet-FL-mCherry (blue) were excited at 517 nm. The spectra were normalized to 530 nm peak to emphasize the FRET signal change. (C) and (E) The fluorescence decay of CyPet (cyan) was fitted by two exponentials giving an average intensity weighted lifetime of 2.36 ± 0.01 ns (mean ± SD; n = 5). The decreased lifetime of CyPet in presence of an acceptor showed an average lifetime of 1.33 ± 0.03 ns (means ± SD; n = 5) for CyPet-GRSMG-mCLIFY (red, C) and 1.07 ± 0.01 ns (means ± SD; n = 5) for CyPet-GRSMG-YPet (blue, E). (D) and (F) The fluorescence lifetime of mCLIFY (cyan, D) and YPet (cyan, F) fitted with two exponentials give an average lifetime of 3.5 ± 0.01 ns (mean ± SD; n = 5) and 3.5 ± 0.001 ns (mean ± SD; n = 5) ns, respectively. The lifetime of the donor for mCLIFY-FL-mCherry (D) decreased to 2.47 ± 0.11 ns (means ± SD; n = 5) and YPet-FL-mCherry (F) to 2.33 ± 0.11 ns (means ± SD; n = 5). The FRET efficincies for all four constructs are given in SI Table 8.

To assess the performance of mCLIFY in FRET applications, we created four constructs using either mCLIFY or YPet as a FRET donor or acceptor (SI Table 8). We compared the changes in FRET signal ratios (acceptor emission (λ = 527 or 610 nm)/donor emission (λ = 475 or 527 nm)) for the four constructs (SI Table 8). As a donor, we paired mCLIFY or YPet with an mCherry acceptor via a ferredoxin-like (FL) linker^11^ and excited at 517 nm. As an acceptor, we linked mCLIFY or YPet to a CyPet donor via a penta-peptide (GRSMG)^38^ linker and excited at 433 nm. The sensitized emission (longer wavelength fluorescence peaks in Figure 6A and B) for CyPet-GRSMG-YPet showed stronger energy transfer than CyPet-GRSMG-mCLIFY (Figure S16A). In contrast, mCLIFY-FL-mCherry showed a slight decrease in energy transfer compared with YPet-FL-mCherry (Figure S16B).

We also determined the FRET efficiencies of these constructs by measuring and comparing the change in donor fluorescence lifetime in the presence and absence of acceptor (Figure 6C-F). The FRET efficiencies obtained from fluorescence lifetime changes (SI Table 8) were consistent with the sensitized emission peaks, yielding FRET efficiencies (%) of 33.51 ± 3.30 (mean ± SD; n = 5) for YPet-FL-mCherry, 29.55 ± 3.23 (mean ± SD; n = 5) for mCLIFY-FL-mCherry, 54.69 ± 0.66 (mean ± SD; n = 5) for CyPet-GRSMG-YPet and 43.68 ± 1.29 (mean ± SD; n = 5) for CyPet-GRSMG-mCLIFY.

## Discussion

In this study, we combined structural biology, solution biophysics, and single molecule approaches to achieve a more comprehensive understanding of the structural and functional mechanisms of a newly created, circularly permuted, and monomeric variant of the commonly used YPet called mCLIFY, which retains all the advantageous photophysical properties of YPet. Part of our motivation for choosing YPet as a template to create mCLIFY was its brightness, long fluorescence lifetime in vivo and resistance to environmental changes, thus making it ideal as either a donor or acceptor for FRET biosensors. mCLIFY shares the desirable characteristics with YPet including an insensitivity to the environment as reflected from its pKa (5.52) and negligible change in the fluorescence lifetime up to 2 M of KCl or NaCl or in the presence of PEG crowding agents. In comparison to mCLIFY, the fluorescence lifetime of YPet did show more variability with PEG concentration. This may be attributed to hydrophobic interactions causing dimer formation or YPet might directly interact with PEG.

Other than YPet, most of FPs exhibit fluorescence lifetimes of less than 3 ns in the green-yellow spectrum (SI, Table 1), except for citrine (3.6 ns) which is less photostable^39^. In comparison, mCLIFY and YPet show long fluorescence lifetimes of 3.44 ns (SI, Table 1). YPet has outperformed most previously reported YFP variants in brightness, photostability and pH-insensitivity, but exhibits concentration dependent dimerization. Heppert *et. al.*^16^ compared some of the brightest green-yellow FPs and found that monomeric mYPet (generated solely with the A206K mutation) is brighter than mNeonGreen but shows poor photostability in vivo. Botman *et. al.*^17^ found that the brightness of the yeast codon optimized FP ymYPet (A206K, F208S, E231L, N234D) is monomeric but considerably dimmer than YPet.

Albeit a monomer in the asymmetric unit, our crystal structure of mCLIFY is the closest approximation currently available for the elusive structure of YPet. For Venus, a crystallographic dimer interface occurs, consistent with the dimer observed in solution^18^. Each monomer of Venus contributes three hydrophobic amino acids that underlie this dimerization interface. In contrast, the data show that Lys40 and Phe42 in the mCLIFY structure, do not create a high-affinity intermolecular interaction (Fig. 2B) and establish that for mCLIFY these interactions also do not exist in solution via extensive SV-AUC and anisotropy experiments. These AUC results also confirm that replacing Ala40 with lysine effectively prevents the self-association of mCLIFY monomers with the large side chains of Lys55 and Phe42 preventing Leu55 from interacting with the other monomer as is observed with Leu221 in Venus. These steric clashes at the dimerization interface prevent these intermolecular interactions even at higher micromolar concentrations.

Our structural findings reveal that the enhanced photophysical properties of the mCLIFY and YPet chromophore are due to the large side chain of Leu58 which directly abuts Tyr37 while making a tight network with surrounding residues. This network positions the phenolic moieties of the Tyr37 and the chromophore in a more parallel arrangement, restricts the chromophore position and mobility, and potentially changes the chromophore’s environment by excluding water molecules in the nearby cavity. This rigidification of the chromophore is expected to enhance its quantum yield and extinction coefficient^40^ as was observed.

Fluorescence quantum yield and long fluorescence lifetime are critical characteristics in optimizing FPs. Here we used the crystal structure of mCLIFY to rationalize the stepwise improvements of QY, *ε*_max_, brightness, and fluorescence lifetime relative to Venus. The systematic mapping of the differences between mCLIFY and Venus sequences resulted in the generation of several mutants that together revealed that the substitutions V58L and L147V (chromophore interacting residues) improved both the QY and *ε*_max_ and consequently improve the brightness by nearly ~30%. Nguyen et al.^8^ found that Leu224 and Phe208 (in the YFP3 sequence) are responsible for the brightness of YFP3. We observed that the corresponding residue Leu58 in mCLIFY increases the brightness by creating more restricted and parallel π-π stacking between Tyr37 and the chromophore. Moreover, the S42F mutation at the dimer interface did not participate in self-association in the absence of Ala40 and thus did not demonstrate any improvement in QY and *ε*_max_ relative to the negative control, mCLIFY^**Venus**^. Since the excited state fluorescence lifetime of a fluorophore correlates with its fluorescence quantum yield, we also showed a positive correlation between these two parameters of mCLIFY mutants.

In contrast to the concentration-dependent behavior of YPet, our data demonstrates that with both purified protein and in the higher effective concentrations of the bacterial cell lysate, mCLIFY is monomeric. Time-resolved anisotropy of purified proteins and spatially-resolved steady-state fluorescence anisotropy (<*r*>) of cells expressing FPs have also been previously used to investigate their oligomeric state^35–37^. Working under monomeric conditions with < 20 nM purified protein for mCLIFY and YPet, well below *K*_D_^1–2^ = 3.4 μM for YPet dimerization, both <*r*> and rotational correlation time for YPet and mCLIFY were similar. While mCLIFY showed no significant change over the same range of concentrations, anisotropy decreased markedly with increasing YPet concentrations to 7 μM. Moreover, the difference in <*r*> between mCLIFY and YPet was also evident from the steady-state anisotropy of the two FPs imaged in *E. coli*. Inasmuch as the fluorescence anisotropy of the larger dimer would be expected to increase with a slower rotational correlation, we observed the opposite suggesting and consistent with the observation that YPet exhibits homo-FRET. This decrease in <*r*> has also been reported for other FPs that naturally dimerize or are purposefully oligomerized to exhibit homo-FRET^35–37^. YPet exhibits a mere 13 nm Stokes shift and 40 nm of overlap between its excitation and emission spectra leading to expectation of strong homo-FRET in the dimer. Therefore, hetero-FRET detectability and efficiency may be influenced if the FP is binding to an adjacent FRET pair and experiencing homo-FRET^35–37^.

The ability of FPs to self-associate is a critical consideration when selecting FRET probes, as this property may lead to erroneous results. Absence of dimerization is key to accurate interpretation of changes in FRET efficiency. In two different FRET pair experiments, the utility of mCLIFY as an excellent donor and acceptor was demonstrated. The slight decrease in FRET efficiency observed for mCLIFY-FL-mCherry relative to YPet-FL-mCherry may be due to different relative spatial orientations of the chromophores in the two constructs. Meng *et. al.*^38^ reported similar findings where they hypothesized that when only the donor or acceptor of a FRET pair was circularly permuted, the distance between the donor and acceptor molecule decreased, yet the FRET ratio signal decreased implicating the chromophore orientation in these changes. The decrease in the FRET efficiency of CyPet-GRSMG-mCLIFY compared to CyPet-GRSMG-YPet, where the donor and acceptor may dimerize, is likely due to monomeric mCLIFY preventing a physical interaction between the donor and acceptor along with the potential alterations in chromophore orientations. Nguyen et al.^8^ designed an improved FRET pair, CyPet-YPet coupled with a 5-amino-acid short linker, that displayed a dramatic 20-fold ratiometric FRET signal change compared to a 3-fold change for their parental constructs (CFP-YFP). Furthermore, Ohashi et. al.^15^ pointed out that most of the signal enhancement reported by Nguyen et al. seemed to be caused by binding of YPet to CyPet within the tethered construct. This effect was substantially reduced when they incorporated the monomerizing A206K mutation^15^. In the current work, the difference in the emission ratios obtained for CyPet-GRSMG-YPet and CyPet-GRSMG-mCLIFY (SI, Fig. 11) is qualitatively consistent with the observations of Ohashi *et. al*.. Despite the widespread use of YPet, our work shows that utilizing mCLIFY as a FRET donor or acceptor provides more accurate interpretation of FRET changes while eliminating any false positive energy transfer in FRET assays owing to dimerization.

Integrating different approaches in creating and characterizing the novel circularly permuted and monomeric mCLIFY, afforded us the opportunity to obtain some crucial missing details about the structural and functional mechanism of the popular YPet. Our findings identify potential tunable structural properties for the development of new variant FPs in the future while providing users with mCLIFY, a robust and monomeric substitute for YPet.

## IN MEMORIUM

The acronym mCLIFY describes our new fluorescent protein and honors Clifford Brody, the husband of Dr. Jody A. Dantzig, who lost his battle with signet ring cell carcinoma while this study was taking place.

## METHODS

### Chemicals:

All synthetic codon-optimized dsDNA fragments (g-blocks), were purchased from Integrated DNA Technologies (IDT). All enzymes for subcloning were from New England Biolabs. Crystallization solutions were purchased from Hampton Research. All other chemicals were from Fisher Scientific, Sigma Aldrich, or Agilent Technology.

### Plasmid Construction:

The sequences for all the constructs in this study are provided in supporting information. Codon-optimized dsDNA sequences (Integrated DNA Technologies, IDT) for in either *E. coli* (YPet, CyPet) or mammalian cells (mCLIFY) were subcloned into a pET24b(+) vector (Novagen, Cat. No. 69750–3) containing the kanamycin resistance gene using *NheI* and *XhoI* restriction sites and an in-frame C-terminus hexahistidine tag (6XHis-tag) incorporated to facilitate protein purification. Similarly, full-length sequences for YPet and mCLIFY for structural studies and mCLIFY variants were subcloned into pET24b(+) vectors containing an in-frame N-terminal 6XHis-tag and TEV protease cleavage site prior to the protein of interest using *NheI* and *XhoI* restriction sites.

To create FRET pairs using YPet and mCLIFY as donor molecules, we started with a YPet^short^-FL-mCherry plasmid (FL denoting ferrodoxin-like) provided by Dr. Carsten Grashoff. This variant lacks the flexible eleven C-terminal amino acids (GITEGMNELYK) known to unfold when force is applied to the FRET sensor^11^. To create the desired constructs, we subcloned the FRET pair into a pET24b(+) vector using *NdeI* and *XhoI* restriction sites. To create the mCLIFY-FL-mCherry construct, a N-termini fragment (mCLIFY-FL-partial-mCherry) of the gene was synthesized and cloned into pET24b(+) using the *NdeI* and *XhoI* restriction sites, then in subsequent cloning, a synthetic C-termini fragment (remainder-partial-mCherry) was inserted between the *AhdI* and *XhoI* sites. Finally, for the acceptor constructs, CyPet- (GRSMG)-mCLIFY and CyPet-(GRSMG)-YPet were subcloned into the pET24b(+) vector using *NheI* and *XhoI* restriction sites.

### Protein Expression:

All constructs were transformed into *E. coli* Arctic (DE3) competent cells (Agilent Technologies Cat. No. 230192) using standard heat shock transformation methods. Overnight cultures were grown from a few colonies in LB medium containing 30 μg/ml kanamycin at 37°C with shaking (247 rpm). For preparative protein expression, 300–500 ml of LB broth with appropriate antibiotic selection was inoculated with 5 ml of the initial cultures and shaken continuously at 37°C. Protein expression was induced with isopropyl β-D-1-thiogalactopyranoside (IPTG) to a final concentration of 0.5 mM when the culture reached OD_595_ ≈ 0.4 and shaken for ~15–16 h at 25°C under low light conditions to ensure complete folding and maturation of the chromophore. The cells were harvested before they reached the stationary phase and death.

### Protein Purification:

Harvested cell pellets were resuspended in 10 ml lysis buffer (50mM NaH_2_PO_4_, 300 mM NaCl, 10 mM Imidazole, pH 8.0) containing one tablet of EDTA-free protease inhibitor cocktail (Sigma-Aldrich, Cat. No. 11873580001). Cells were lysed by sonication for 90 seconds and the lysate clarified by centrifugation at 10,000 rcf for 35 minutes. The supernatant containing soluble protein was mixed with 5 ml HisPur cobalt resin (Thermo Scientific) pre-equilibrated with lysis buffer and incubated at 4°C for 1 h with end-over-end rotation. The suspension was poured into a gravity flow column and washed with 5–10 ml of wash buffer (50mM NaH_2_PO_4_, 300 mM NaCl, 20 mM Imidazole, pH 8.0). His-tagged proteins were eluted with elution Buffer (50mM NaH_2_PO_4_, 300 mM NaCl, 400 mM Imidazole, pH 8.0). The purified protein was dialyzed into phosphate-buffered saline (PBS) pH 7.4 with 0.2 mM tris(2-carboxyethyl) phosphine (TCEP), snap-frozen in liquid N_2_, and stored at −80°C.

For structural studies, proteins were further purified by FPLC (AKTA Pure, Cytiva Life Sciences) using a Superdex 200 10/300 GL size exclusion column (SEC, Cytiva Life Sciences) pre-equilibrated in PBS buffer with additional 150 mM NaCl. Samples were isocratically eluted at a flow rate of 0.3 ml/min with 0.5 ml fractions while monitoring absorbance at 280 nm (Abs_280 nm_) for the presence of protein, Abs_517nm_ for the YFP chromophore, and fluorescence emission at 530 nm (Em_530nm_) following excitation at 517 nm (Ex_517nm_) to isolate the peak containing a homogeneous population of folded protein with mature chromophores. Fractions were collected within the peak that fulfilling these criteria (Fig. S17), pooled and concentrated using an Amicon Ultra-4 centrifugal filter with 10 kDa cutoff (Millipore-UFC801024). The N-terminal 6XHis-tag was cleaved by incubation overnight at 4°C with TEV protease (1:100; TEV protease: FP). After cleavage, samples were passed through the cobalt column again using the protocol above to capture liberated 6XHis-tags. YPet and mCLIFY eluent were concentrated to ~14 mg/ml and washed 5 – 6 times with 5 ml of 10 mM HEPES buffer pH 7.4 using the Amicon Ultra-4 centrifugal filters. The purified protein was dialyzed into 10 mM HEPES buffer with 0.2 mM TCEP, snap-frozen in liquid N_2_, and then stored at −80°C. The purity of the final samples was confirmed by SDS-PAGE analysis (Fig. S18).

### Photophysical Properties:

#### Absorption and Emission Spectra:

Individual fluorescent proteins and the FRET pairs were diluted to 1 μM in PBS to obtain absorption, and emission spectra (Fig. 1B, S7C, S7D and S19). Absorbance was measured from 400 – 650 nm using a 1 nm step size with a Cary60 UV-Vis spectrophotometer (Agilent Technology). Excitation and emission spectra were obtained using a variable wavelength Photon Technology Instruments/Horiba QuantaMaster fluorometer with dye laser excitation. Fluorescence emission spectra were collected over the range of 500–700 nm with Ex_488nm_ using a step size of 0.5 nm and excitation and emission slits at 2 nm and 4 nm, respectively (Fig. 1B, S7D). YPet-FL-mCherry, mCLIFY-FL-mCherry were excited at 517 nm and CyPet-GRSMG-YPet, CyPet-GRSMG-mCLIFY were excited at 433 nm (Fig. 6A and 6B).

#### *Extinction Coefficient* (*ε*_max_):

To determine extinction coefficients, absorption spectra (λ = 250–650 nm), were measured for protein concentrations in the OD_517_ range of 0.4 – 0.6 then diluted 50% with 2 M NaOH (1 M final concentration) to denature the protein. Spectra of the denatured protein showed complete loss of Abs_517nm_ and appearance of the 445 nm peak associated with the stable and mature primary green fluorescent protein (GFP) type chromophore that is resistant to denaturing following maturation and known to have *ε*_445nm_ = 44,000 M^−1^cm^−1^ used to calculate mature chromophore concentration^41^ using Beer-Lambert’s law. Based on these concentrations, *ε*_517_ were calculated from the maximum absorbance for YPet and mCLIFY at λ_517_ (Fig. 1C).

#### Quantum Yield (QY):

To determine the fluorescence quantum yield, we used low concentration samples (OD_488_ <0.1) to minimize inner filter effects which would attenuate the fluorescence signal. YPet was used as the standard with its 0.77 reported quantum yield^8^ ($m_{std}$) due to its similar spectral properties to mCLIFY and the mutants. A series of five samples were prepared by serial dilution with PBS buffer. Emission spectra (λ = 500–650 nm) were obtained with 488 nm excitation. All spectra were recorded with a 0.5 nm step size and excitation and emission slits at 2 nm and 4 nm, respectively. The integrated fluorescence intensity from 500 – 650 nm was plotted against absorbance at 488 nm (Fig. 1D). The slope of the linear fit for YPet was used to obtain the quantum yield of unknown FP using the equation:

(1) $$QY_{FP}=QY_{std}\left(\frac{m_{FP}}{m_{std}}\right)\left(\frac{\eta_{FP}^{2}}{\eta_{std}^{2}}\right)$$

Where, $QY_{FP}$, and $QY_{std}$ are the quantum yields of the unknown FP and YPet respectively. $m_{FP}$ and $m_{std}$ are the slopes of the linear fit of unknown FP and YPet. Since PBS was used for the standard and sample, $\eta_{FP}^{2}=\eta_{std}^{2}$ and represent the index of refraction.

#### pKa:

To determine pH dependence, absorbance (λ = = 250–650 nm) and emission (λ = 500–650 nm) spectra of mCLIFY and YPet across a range of pH values (pH 3.0 – 5.5, 100 mM citric acid/Na citrate, pH 6–8, 100 mM KH_2_PO_4_/Na_2_HPO_4_ and pH 8.5–10.0, 100 mM NaOH/Glycine) were measured. To evaluate the pKa, intensity of emission spectra integrated over 510 to 650 nm was plotted against pH for both mCLIFY and YPet. pKa values (Fig. S3) for mCLIFY (pKa = 5.52 ±0.06, Hill coefficient (*n*_*H*_) = 1.14 ± 0.17 (mean ± SD, n = 4)) and YPet (pKa = 5.47 ± 0.04, ($n_{H}$) = 1.00 ± 0.08 (mean ± SD, n = 4)) were approximated using the following equation:

(2) $$F(pH)=F_{min}+\frac{F_{max}-\;F_{min}}{1+10^{n(pKa-pH)}}$$

Where $F_{min},\;F_{max}$ are the minimum and maximum integrated fluorescence intensities. $n_{H}$ is the Hill coefficient. pKa is defined as the pH value where fluorescence decreases by 50%.

To determine if the decrease of fluorescence intensity observed at low pH values was due to a low pH condition or denaturation, we measured and compared the fluorescence intensity of the FPs at pH ~7.4, 5.0 and at a pH titrated from 5.0 to 7.4. The intensity decrease observed at pH 5.0 recovered by 80–90% in less than a minute when the pH was titrated back to ~7.4. The residual loss of intensity was not further recovered by waiting longer. We found that a small fraction of fluorescence intensity was lost due to protein quenching or denaturation.

#### Bleaching rate:

Two methods were used to determine the bleaching rate. YPet and mCLIFY samples (1 μM) were excited at 473 nm using a diode-pumped solid-state laser (MBL-III-473/1, Opto Engine LLC). Laser power was measured using a power meter (PM100D Thorlabs, Inc) before each measurement. Samples were illuminated in 5-minute intervals, followed by gentle mixing within the cuvette and subsequent absorbance measurements. The recorded optical density values over time for the bleached samples (Fig. S4A, S4B) were fitted with the following equation:

(3) $$OD=A_{0}\;*\;{exp}^{\left(-t\cdot r\right)}$$

where, $A_{0}$ is the initial value, t is the time in seconds and r is the decay rate constant.

Fluorescence intensity measurements were also performed with a range of FP concentrations (100, 50, and 25 nM samples) on a Tecan GENios Pro multifunctional plate reader (GENios; Tecan Trading AG, Salazburg, Austria). 384-well small volume HiBase polystyrene microplates (Greiner Bio-One) were used. Samples were excited at 485 nm and the fluorescence intensity measurements were collected for 2 minutes 25 s for 50 cycles. In each cycle, ten fluorescence intensity measurements were taken in each well for 40 μs illumination periods and averaged. Each sample was thus illuminated for 400 μs per cycle. Samples were shaken at 220 rpm for 5 s before measurements and in between cycles. Background corrections were made by subtracting the fluorescence intensity measured with PBS buffer and the recorded fluorescence intensities were fitted by the following equation:

(4) $$Fluorescence\;intensity=(1-b)+A_{0}\;*\;{exp}^{\left(-t\cdot r\right)}$$

Where, b is the offset, t is the time in seconds and r is the decay rate constant.

The average decay rate of mCLIFY and YPet (Fig. S4E) measured using 473 nm laser was determined to be 7.3 ± 0.55 × 10^−4^ s^−1^ and 6.9 ± 0.59 × 10^−4^ s^−1^ (means ± SDs; n = 5), respectively. The excitation peak for both mCLIFY and YPet is ~517 nm, therefore the decay rate obtained using 473 nm (473 nm excites ~19 % of excitation spectra) was corrected for the excitation wavelength and the laser power (15.05 ± 1.24 mW/16 mm^2^ for mCLIFY and 14.58 ± 0.647 mW/16 mm^2^ for YPet) to compare it with the reported bleaching half time (30.3 s and 58 s at 20 mW/mm^2^) of YPet^42^. Since, the sample volume for each sample was 70 μl, only 70 % of the sample was bleached at each reading using 50 μl cuvette. Thus, the obtained decay was also corrected for the non-illuminated sample volume mixed in before measuring absorption. Correcting for all the mentioned factors, the bleach half times for mCLIFY and YPet in solution calculated under the same conditions are 12.20 and 12.43 seconds, respectively, at 20 mW/mm^2^.

The bleaching times of YPet and mCLIFY were also obtained using the Tecan GENios Pro plate reader set to 485 nm excitation for three different protein concentrations (Fig. S4C, S4D). The average decay rate for mCLIFY (Fig. S4F) was not significantly different with 7.64 ± 0.57 × 10^−4^ μs^−1^ (mean ± SD; n = 5) at 100 nM, 7.27 ± 0.52 × 10^−4^ μs^−1^ (mean ± SD; n = 5) at 50 nM, and 7.67 ± 0.72 × 10^−4^ μs^−1^ (mean ± SD; n = 5) at 25 nM. The average decay rate for YPet (Fig. S4F) was similar with 7.65 ± 1.01 × 10^−4^ μs^−1^ (mean ± SD; n = 5) at 100 nM, 7.26 ± 0.67 × 10^−4^ μs^−1^ (mean ± SD; n = 5) at 50 nM, and 7.64 ± 0.87 × 10^−4^ μs^−1^ (mean ± SD; n = 5) at 25 nM. Comparing both the FPs under same condition obtained above shows similar bleaching rates.

#### Time-resolved measurements:

Fluorescence lifetime, anisotropy (*in vitro* and in *E. coli* cells) and FRET measurements were performed on a MicroTime 200 confocal fluorometer (PicoQuant, Germany). All measurements were done using Nunc Lab-Tek 200 μL chambers (ThermoFisher-155411) with borosilicate cover slip bottoms. For *in vitro* measurements these chambers were passivated by treatment with 50 % (w/v) PEG-8000 solution, incubated at room temperature for 2–3 hours, followed by 3–4 washes with PBS buffer.

For the YFP fluorescence measurements on the MicroTime 200 fluorometer, samples were prepared in pH 7.4 PBS buffer. The samples were excited at 484 nm (YPet, mCLIFY, mutants) using a pulsed diode laser (LDH-D-TA-484, PicoQuant) operating at 20 MHz, *via* an excitation dichroic filter ZT440–445/484–491/594 rpc-UF3 (Chroma Technology) and an Olympus UPLanSApo 60x/1.20W water objective lens. Fluorescence signals were detected for at least 600 s with a single photon detector (SPCM-AQRH-14-TR) at 16 ps time-correlated resolution through a 50 μm diameter pinhole and ET535/70m, emission filter (Chroma Technology). For FRET measurements, YPet-FL-mCherry and mCLIFY-FL-mCherry samples were excited at 484 nm and the fluorescence emission signals were separated into the donor and acceptor channels with a 585dcxr dichroic filter, a ET535/70m bandpass filter for the donor and a 610 LP long pass filter (all Chroma Technology) for the acceptor channel. For fluorescence lifetime and FRET measurements of CyPet, CyPet-GRSMG-YPet and CyPet-GRSMG-mCLIFY, each sample was excited at 443 nm using a pulsed diode laser (LDH-D-TA-443, PicoQuant) and the signals were separated into donor and acceptor channels with a T525lpxr dichroic filter and 450–480 nm emission bandpass filter (Chroma Technology) and a ET510 LP long pass filter. For time-resolved and steady state FLIM anisotropy measurements, the fluorescence was split into two channels based on polarization using a 50:50 polarizer cube (U-MBF3-Olympus). Emitted photons parallel and perpendicular to the excitation polarization were passed through ET535/70 emission filters into both detector arms.

To calculate the fluorescence lifetime, the recorded TCSPC (***T****ime* ***C****orrelated* ***S****ingle* ***P****hoton* ***C****ounting)* fluorescence decays were fitted using SymphoTime64 software (PicoQuant, Germany) by one or two exponential re-convolution models using the SymphoTime64-generated instrument response functions (IRFs). The number of components and quality of the fit were assessed by the chi-squared (χ^2^) criterion for each fit.


(5) $$y(t)=IRF⊗\sum_{i}\;A_{i}e^{-t/\tau_{i}}+Bkg$$

Where, $A_{i}=$ exponential factor (amplitude), $\tau_{\iota }$ is the exponential decay time constant for the *i*^th^ decay component, respectively, and Bkg is the baseline correction for background after-pulsing, dark counts, and ambient light. ⊗ Indicates the convolution calculated within the fitting routine.

The average intensity weighted fluorescence lifetime was calculated as:

(6) $$\tau_{AvInt}=\frac{\sum_{i}\;\;A_{i}\tau_{i}^{2}}{\sum_{i}\;\;A_{i}\tau_{i}}$$

FRET efficiencies reported in SI Table 8 were calculated using the following equation:

(7) $$E=1-\frac{\tau_{DA}}{\tau_{D}}$$

Where, $\tau_{D}$ is the average lifetime of donor and $\tau_{DA}$ is the amplitude-weighted average lifetime of donor in presence of acceptor calculated using the following equation:

(8) $$\tau_{AvAmp}=\frac{\sum_{i}\;\;A_{i}\tau_{i}}{\sum_{i}\;\;A_{i}}$$

#### Intensity Weighted Steady State Anisotropy with FLIM:

YPet and mCLIFY were expressed in Arctic (DE3) *E. coli* cells under the conditions described above. A sample was obtained prior to IPTG induction, 15 minutes after induction and then every 30 minutes after IPTG induction. Samples were transferred to non-passivated chambers and allowed to settle for 5 mins while the cells adhered to the coverslip bottom. FLIM images of the parallel and perpendicular emissions were obtained via a monodirectional raster scan of an 80 μm X 80 μm region at the surface of the chamber with 208 nm/pixel lateral resolution using the optical configurations described above for YFPs and with reduced laser intensity to avoid saturation, photobleaching and cellular damage. Each full scan was completed within 4 minutes.

The recorded FLIM images were recorded with the SymphoTime64 software using its anisotropy module to obtain and save images of the fluorescence intensities parallel and perpendicular to the excitation in OME.TIF format. ImageJ/Fiji macro programs calculated steady state anisotropy for each pixel ($r_{\text{ss}}$, equation 10).


(10) $$Anisotropy\left(r_{ss}\right)=\frac{I_{∥}-\;G\cdot I_{⊥}}{I_{∥}\cdot \left(1-3L_{2}\right)+2\cdot G\cdot I_{⊥}\cdot \left(1-1.5L_{1}\right)}=\frac{N}{D}$$

Where $I_{∥}$ and $I_{⊥}$ are the background-corrected parallel and perpendicular fluorescence intensities, respectively. Background intensities were determined from the image regions away from bacteria. G (1.035) is the correction factor for the relative detection efficiency between the two detector channels. $L_{1}$ (0.0308) and $L_{2}$ (0.0368) are the correction factors for mixing of the parallel and perpendicular excitation and emissions polarizations by refraction in the objective^43^. N and D are the numerator and denominator at each pixel.

For anisotropy values averaged over spatial areas and for histograms (Fig 5. E and F), the pixel anisotropy values accumulated were weighted by each of their intensities, which is D in equation 10.


(11) $$Weighted\;Anisotropy\left(r_{w}\right)=\frac{\sum_{np}\;r_{ss}\cdot D}{\sum_{np}\;D}=\frac{\sum_{np}\;N}{\sum_{np}\;D}$$

Where np = number pixels in the average.

For plotting anisotropy vs. expressed FP concentration in the cells, the same detection and analyses were performed on images of known concentrations of purified FP in PBS solutions up to the maximal fluorescence intensity observed in FP-expressing *E. coli*, corresponding to ~40 μM FP. These calibration FLIM images were obtained 2.5 μm into the solution above the coverslip surface in PEG-passivated chambers. The concentration of fluorophore was plotted *vs*. the fluorescence intensity (*D*) (Fig. S15C).

#### Ensemble Average Anisotropy:

The effect of concentration and crowding agents on ensemble average anisotropy was determined using Photon Technology Instruments/Horiba QuantaMaster fluorometer with excitation from a xenon arc lamp. The FP samples were prepared in PBS buffer at a concentration ranging from 0.5 μM to 7 μM. Each sample was excited at 484 nm (slit width 2 nm) and polarizers were used to separate the parallel and perpendicular fluorescence emission collected at 530 nm into the two channels. Data points for each sample were collected once per second for 20s and then averaged (Fig. S15A, S15B).

### X-ray Crystallography:

All trials for crystallization were performed using the sitting drop method: a 1:1 mixture of mCLIFY and a reservoir solution is vapor equilibrated against the same reservoir solution. After ~3 weeks incubation at 23°C, crystals appeared in a reservoir containing 0.1 M Sodium citrate tribasic dihydrate pH 5.5, 20% (w/v) PEG4000 and 18% (v/v) 2-Propanol. The crystals were cryoprotected by adding glycerol to the drop to a final concentration of 15% one day before freezing and storage in liquid nitrogen.

Diffraction data for mCLIFY crystals were collected at 100°K and wavelength of 1 Å on beamline FMX at NSLSII (Brookhaven National Laboratory, Upton, NY)^44^. Images were processed using the *fastdp* pipeline software package^(45)^ and MOSFLM^(46)^. Molecular replacement using the biosensor Twitch-2B green fluorescent protein structure (PDB: 6GEL^47^, amino acids 309 to 547) was used as an initial search model with the Phenix software package program PHASER^48^ Models were iteratively refined in reciprocal space with Phenix (which includes rigid body refinement, simulated annealing, energy minimization, TLS, and individual B refinement) and in real space with Coot^49^. All illustrations of the structure were prepared using PYMOL (The PyMOL Molecular Graphics System, Version 2.0 Schrödinger, LLC.

### Sedimentation velocity analytical ultracentrifugation (SV-AUC):

To investigate dimerization of the FPs, sedimentation velocity-analytical ultracentrifugation (SV-AUC) experiments were performed at 20°C with an XL-A analytical ultracentrifuge (Beckman) and a TiAn60 rotor with two-channel epon charcoal-filled centerpieces and quartz windows. Experiments were performed in PBS with FP protein concentrations of 2 – 40 μM monomer as calculated using experimental extinction coefficients provided in SI, Table 2. Sedimentation velocity profiles were collected every 30 s for 200 boundaries at 40,000 rpm. Data were fit using the *c(s)* distribution variant of the Lamm equation model, as implemented in the program SEDFIT^50^. After optimizing meniscus position and fitting limits, the sedimentation coefficient(s) and best-fit frictional ratio (*f/f*_*0*_) was determined by iterative least squares analysis. Sedimentation coefficients were corrected to *S*_20,w_ based on the calculated solvent density (ρ) and viscosity (η) derived from chemical composition by the program SEDNTERP^51^. Figures were prepared using the program GUSSI^52^. Calculated hydrodynamic properties for atomic models were determined using WinHYDROPRO^53^.

### Size-Exclusion chromatography in-line with small angle X-ray scattering and multiangle light scattering (SEC-SAXS-MALS).

Data were collected at the SIBYLS beamline of the Advanced Light Source Light Source II (Berkeley, CA). Data was collected at a wavelength of 1.0 Å in a three-camera configuration yielding accessible scattering angles where 0.006 < q < 3.0 Å^−1^, where q is the momentum transfer (defined as q = 4π sin(θ)/λ), λ is the X-ray wavelength and 2θ is the scattering angle; data out to q < 0.3 Å^−1^ were used in subsequent structural analyses. 100 μl of purified 260 μM YPet or 230 μM mCLIFY in PBS were injected and eluted isocratically from a Shodex 804 sizing column (Showa Denko American, Inc., New York, NY, USA) equilibrated in PBS, at room temperature. Eluent from the column flowed into a 1 mm capillary for subsequent X-ray exposures at 1 s intervals. Plots of intensity from the forward scatter closely correlated to in-line UV and refractive index (RI) measurements.

Absolute molar mass of the proteins was determined in-line using multi-angle light scattering. Light scattering from the column eluent was recorded at 18 different angles using a DAWN-HELEOSII MALS detector (Wyatt Technology Corp.) operating at 658 nm. Protein concentration of the eluent was determined using an in-line Optilab T-rEX Interferometric Refractometer (Wyatt Technology Corp.). The weight-averaged molar masses of species within defined chromatographic peaks were calculated using the ASTRA software version 6.0 (Wyatt Technology Corp.), by construction of Debye plots (KC/Rθ versus. sin^2^[θ/2]) at one second data intervals. The weight-averaged molar mass was then calculated at each point of the chromatographic trace from the Debye plot intercept and an overall average molar mass was calculated by weighted averaging across the peak.

### Small Angle Xray Scattering (SAXS) Analysis.

SVD-EFA (**S**ingular **V**alue **D**ecomposition-**E**volving **F**actor **A**nalysis) analysis of the SEC-SAXS data sets was performed as previously described, as implemented in the program RAW^29^. Buffer subtracted profiles were analyzed by singular value decomposition (SVD) and the ranges of overlapping peak data determined using evolving factor analysis^54^. The determined peak windows were used to identify the basis vectors for each component and the corresponding SAXS profiles were calculated. When fitting manually, the maximum diameter of the particle (D_max_) was incrementally adjusted in GNOM^55^ to maximize the goodness-of-fit parameter, to minimize the discrepancy between the fit and the experimental data, and to optimize the visual qualities of the distribution profile. Low resolution shapes were reconstructed *ab initio* from solution scattering data using the program GASBOR^33^. The number of beads (amino acids) used in the calculation was prescribed based on protein length. Ten independent calculations were performed for each data set using default parameters. With the dimeric YPet data, a 2-fold symmetry restraint was employed. The models resulting from the independent runs were superposed by the program SUPCOMB^56^ based on the normalized spatial discrepancy (NSD) criterion. The ten independent reconstructions were then averaged and filtered to a final consensus model using the DAMAVER suite of programs^57^. Hybrid bead-atomistic modeling of fluorescent proteins was performed using the program CORAL^34^, where the known structure was fixed, and inventory residues not resolved by X-ray crystallography were modeled as coarse grain beads. Ten independent calculations for each protein were performed and yielded comparable results. The final models were assessed using the program FoxS^58^. The models were rendered using the program PYMOL.

## Extended Data

**Extended Figure1.**
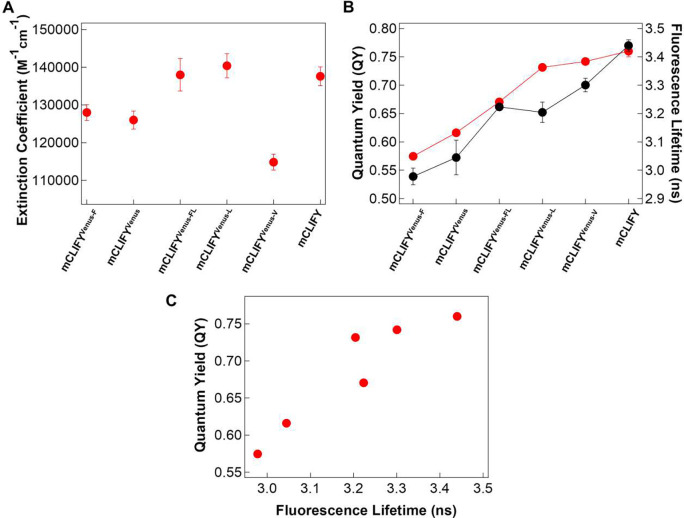
Photophysical properties of mCLIFY mutants. (A) Extinction coefficients (*ε*_max_) of denatured mCLIFY mutants. (B) Quantum yield and fluorescence (described in Methods) for each mutant. - (C) A linear and positive correlation between fluorescence lifetime and quantum yield of mCLIFY mutants.

## Data Availability

Data are available in the manuscript or the supplementary material. Materials are available upon request to corresponding authors.
